# Why and how can HTA support evidence-informed health policymaking?

**DOI:** 10.1093/eurpub/ckac129.085

**Published:** 2022-10-25

**Authors:** I Gutiérrez-Ibarluzea, N Ibargoyen-Roteta, G Benguria-Arrate, L Galnares-Cordero

**Affiliations:** Basque Office for Health Technology Assessment, La Fundación Vasca de Innovación e Investigación Sanitarias, Barakaldo, Spain

## Abstract

**Issue:**

Health care systems actors are in need to take decisions every day. The basis for a good decision is to consider all the factors, the value of the action, the values that support a “go” or “not go” decision and the intended and unintended consequences that those decisions could entail. Furthermore, understanding decisions implies knowing which the context and the circumstances that surround decisions are.

**Description of the issue:**

HTA should cover all those aforementioned aspects and make it along the life cycle of health technologies. Thus, it should be the pivotal element that health systems should look at when making decisions. Nevertheless, this is not the case in all the countries/regions or health systems. Empirical research (facts) and normative inquiry (values) are not consistently considered and holistic approaches to the determination of value are lacking.

**Results:**

Policymakers, health care systems and HTA bodies are differentially approaching to value determination, and, in most cases, this is not explicitly shared with the stakeholders concerned. The consideration of the implication of life cycle concept is not well addressed and decisions are mainly focused on introduction and implementation, meanwhile decisions on deletion or modification of use are lacking, although relevant for health systems sustainability. Mostly systems failed by individual determination of technologies’ value without considering that they are used on systems in combination to other technologies, by different professionals, applied to different patients and within different contexts.

**Lessons:**

There is a need for a holistic approach of value determination. Likewise, the life cycle concept offers a unique opportunity to promote proactive actions in improving added value generation for technologies under development. Similarly, the assessment of value once solutions are introduced is substantially a way to improve systems’ outcomes and their efficiency.

